# Complications and functional outcomes of restorative proctocolectomy for ulcerative colitis in the elderly

**DOI:** 10.1186/1471-2482-13-S2-S9

**Published:** 2013-10-08

**Authors:** Gianluca Pellino, Guido Sciaudone, Giuseppe Candilio, Antonio Camerlingo, Rosa Marcellinaro, Federica Rocco, Serena De Fatico, Silvestro Canonico, Francesco Selvaggi

**Affiliations:** 1 Unit of General and Geriatric Surgery, Second University of Naples, Naples, Italy; 2 Department of Internal Medicine, Surgical, Neurological Metabolic Disease and Geriatric Medicine, Second University of Naples, Italy

**Keywords:** Ileopouch-anal anastomosis, IPAA, ulcerative colitis, elderly patients, restorative proctocolectomy, quality of life

## Abstract

**Background:**

Restorative proctocolectomy with ileopouch-anal anastomosis (IPAA) is the treatment of choice for intractable or complicated ulcerative colitis (UC). Debate exists concerning outcomes of IPAA in the elderly and literature data are scarce. We report our experience of IPAA in older population.

**Methods:**

We gathered data on a prospective database of patients undergoing IPAA for UC over 70 years of age in our Unit from January 1990 through January 2010. Patients were compared with randomly selected younger controls on a 1:3 ratio. Patients underwent IPAA in 2 or 3 stages. Demographical data, disease characteristics, comorbidities, concomitant medications, peri-operative management, intra- and post-operative complications were analyzed. Function and quality of life were assessed by clinical visit and Inflammatory Bowel Disease Questionnaire 1 and 3 years after ileostomy takedown.

**Results:**

Twenty-seven elderly patients underwent IPAA for UC in the study period; these were compared with 81 younger controls. The former had more comorbidities and higher ASA score. All patients underwent loop-ileostomy closure. There were no differences between groups concerning the rate of major complications, but elderly patients more frequently had nuisances due to stoma output. Younger patients experienced significantly more episodes of small bowel obstruction. No significant differences in bowel control and health-related quality of life was observed, except for an higher rate of elderly patients taking antidiarrhoeals at 1-year follow-up; this observation was not confirmed at 3-year follow-up. A minimal decrease in continence was observed, but this did not affect overall satisfaction.

**Conclusions:**

IPAA can be safely offered to selected elderly UC patients who are strongly motivated and with no clinical disturbances of continence. In experienced hands no differences are likely to be expected concerning complications, quality of life and function. Results are stable with time and comparable to those of younger patients.

## Background

Restorative proctocolectomy with ileo-pouch-anal anastomosis (IPAA) is the procedure of choice for complicated or refractory ulcerative colitis (UC) [1,2]. Recent data suggest that IPAA is suitable also in patients in the elderly; however, in published series patients are often classified as "in the elderly" when aged >45 years [2-5]. Furthermore, debate exists concerning peri-operative morbidity, bowel function and quality of life in this subgroup of patients.

Aim of our study was to compare the outcome of IPAA in patients undergoing surgery >70-year-old with those aged <70 with respect to perioperative complications and function over time, and to demonstrate that this procedure is safe and effective also in the elderly.

## Methods

We gathered data from the prospective database of patients undergoing IPAA for UC aged >70 years in our Unit from January 1990 through January 2010. Patients were compared with randomly selected younger controls on 1:3 ratio. Two groups were established on the basis of chronological age: group A ≥70 years, group B < 70 years. Life expectancy in Italy is approximately 78.90 (78.70-79.10) years in male and 83.90 (83.70-84.10) in female population, and similar data are found across Europe [6]. We assumed 70 years as a threshold in our analysis as a person is usually unable to cope with work or is retired by the age of 70. Demographical data, disease characteristics, comorbidities, concomitant medications, peri-operative management, intra- and post-operative complications were thoroughly collected.

### Surgical pathway

Surgery was performed in 2 stages (1: proctocolectomy with IPAA and loop-ileostomy; 2: ileostomy takedown) or in 3 stages (1: subtotal colectomy with terminal ileostomy; 2: completion proctectomy, IPAA and loop-ileostomy; 3: ileostomy takedown). Completion proctectomy with IPAA was performed 6-7 months after colectomy; loop-ileostomy takedown was performed 2-3 months after IPAA. Patients undergoing 1-stage and 2-stage modified IPAA were excluded from the study.

### Postoperative evaluation

Perioperative complications and mortality were defined as complication and decease occurring between surgical intervention and discharge. Function was assessed during office visit 6 months after ileostomy closure then yearly for at least 3 years. Visits consisted of clinical exam, including digital examination of the IPAA and flexible pouchoscopy. All patients completed Inflammatory Bowel Disease Questionnaire (IBDQ) to assess health-related quality of life (HRQoL) after ileostomy takedown [7,8].

### Statistical analysis

Results are expressed as or mean ± SD unless otherwise indicated. For continuous parameters *t*-test was used. Comparisons between categorical variables were analyzed using the Fisher's exact test. P <.05 was considered statistically significant.

## Results

Twenty seven patients aged over 70 years underwent IPAA in the study period and were included in Group A; they were compared with 81 younger controls (Group B). Details of patients and surgical procedure are reported in table 1. Group A patients had more comorbidities and higher ASA score. All patients underwent loop-ileostomy closure. No detrimental effect was observed, except for two patients excluded from 3-year follow-up: one dead because of an acute myocardial infarction 2 years after IPAA, another one needed pouch excision because of intractable pouchitis.

**Table 1:** Characteristics of Patients by Age

Characteristics	Group A >70 (n 27)	Group B <70 (n 81)	*P value*
mean (± SD) age, years duration of disease, years	77.5 (±3.1) 14.3 (±8.1)	29.9 (±10.6) 8.9 (±7.4)	**< 0.0001** 0.07

BMI, mean (± SD)	20.5 (±3.6)	21 (±5.7)	0.22

sex, n (M/F)	12/15	32/49	0.66

use of steroids at time of surgery, n (%)	13 (48.1)	31 (38.3)	0.37

use of azathioprine at time of surgery, n (%)	1 (3.7)	0 (0)	0.25

use of other-than-anti-UC drugs, n (%)	16 (59.3)	31 (38.3)	0.07

comorbidities, n (%)	24 (88.9)	21 (17.3)	**<0.0001**

hypoalbuminemia, n (%)	8 (29.6)	16 (19.7)	0.29

ASA score ≥ III, n (%)	9 (33.4)	5 (6.2)	**0.001**

3-stage procedure, n (%)	5 (18.5)	12 (14.8)	0.76

hand-sewn anastomosis, n (%)	9 (33.4)	31 (38.3)	0.82

SD: standard deviation

BMI: body mass index

UC: ulcerative colitis

ASA: American Society of Anesthesiologist

Complications are depicted in table 2. There was no intraoperative or perioperative mortality. No differences between groups concerning the rate of major complications were observed. Elderly patients suffered more frequently for dehydration due to stoma output; this required readmission in one Group A patient. Younger patients experienced significantly more episodes of small bowel obstruction, but only in one case surgery was advocated. Function and HRQoL over time are reported in table 3. At 1-year follow-up elderly patients were taking significantly more antidiarrhoeal medications, but this observation was not confirmed at 3-year follow-up. A minimal decrease in continence was observed, but this did not affect overall satisfaction and HRQoL.

**Table 2:** Postoperative Complications by Age

	Group A	Group B	*P*
	>70 (n 27)	<70 (n 81)	*value*
**Perioperative morbidity**			
Minor			
Prolonged ileus	7 (25.9)	24 (29.6)	NS
Electrolytes loss from stoma output	14 (51.8)	19 (23.4)	**0.008**
Surgical wound infection	1 (3.7)	2 (2.5)	NS

Major			
Hemorrhage	0 (0)	2 (2.5)	NS
Anastomotic leak	1 (3.7)	3 (3.7)	NS
Compartment syndrome of lower limb	0 (0)	1 (1.2)	NS

**Systemic morbidity**			

Asymptomatic pancreatitis	1 (3.7)	1 (1.2)	NS

Symptomatic portal vein thrombosis	0 (0)	1 (1.2)	NS

PE	1 (3.7)	1 (1.2)	NS

UTI	1 (3.7)	3 (3.7)	NS

PI	2 (7.4)	4 (4.9)	NS

AMI	0 (0)	1 (1.2)	NS

**Late postoperative morbidity**			

SBO	1 (3.7)	17 (20.1)	**0.04**

Anastomotic stricture	3 (11.1)	6 (7.4)	NS

Chronic/recurrent pouchitis	2 (7.4)	7 (9.8)	NS

Pouch-vaginal fistula	0/15 (0)	1/49 (2)	NS

Symptomatic retained rectal stump	0 (0)	1 (1.2)	NS

Twisting reservoir	1 (3.7)	0 (0)	NS

NS: not significant

PE: pulmonary embolism

UTI: urinary tract infection

PI: pulmonary infection

AMI: acute myocardial infarction

SBO: small bowel obstruction

**Table 3:** Function and Quality of Life by Age at 1-Year and 3-Year Follow-Up

	Group A >70 (n 27)	Group B <70 (n 81)	*P* *value*
**1-year follow-up**	**n (%)**	**n (%)**	

***Function***			

Mean stool frequency per day (±SD)	4.6 (±2.1)	4.8 (±2.3)	NS

Patients with night evacuation	7 (25.9)	20 (24.7)	NS

Urgency	2 (7.4)	6 (7.4)	NS

Incontinence during day	1 (3.7)	2 (2.5)	NS

Incontinence during night	2 (7.4)	2 (2.5)	NS

Impaired discrimination	6 (22.3)	15 (18.5)	NS

Pad	7 (25.9)	18 (22.3)	NS

Antidiarrhoeals	14 (51.9)	23 (28.4)	**0.03**

***IBDQ***			

Excellent/Good (>150)	16 (59.3)	52 (64.2)	NS

Regular (101-150)	10 (37)	27 (33.3)	NS

Bad (<101)	1 (3.7)	2 (2.5)	NS

	Group A*	Group B*	***P***

	**>70 (n 26)**	**<70 (n 80)**	***value***

**3-year follow-up**	**n (%)**	**n (%)**	

***Function***			

Mean stool frequency per day (±SD)	4.8 (±2.2)	4.9 (±1.9)	NS

Patients with night evacuation	7 (26.9)	21 (26.2)	NS

Urgency	3 (11.5)	9 (11.2)	NS

Incontinence during day	1 (3.8)	2 (2.5)	NS

Incontinence during night	2 (7.7)	2 (2.5)	NS

Impaired discrimination	7 (26.9)	17 (21.2)	NS

Pad	6 (23)	19 (23.7)	NS

Antidiarrhoeals	12 (46.1)	24 (30)	NS

***IBDQ***			

Excellent/Good (>150)	14 (53.8)	48 (60)	NS

Regular (101-150)	11 (42.3)	30 (37.5)	NS

Bad (<101)	1 (3.9)	2 (2.5)	NS

*One patient of each group was lost at 3-year follow-up: 1 Group A patient needed pouch excision for intractable pouchitis; 1 Group B patients died for myocardial infarction 2 years after surgery.

## Discussion

IPAA is the mainstay treatment for intractable or complicated UC. It cures UC removing the entire diseased mucosa and controls some extra-intestinal manifestations. The risk of cancer is eliminated, provided that dysplasia or cancer are not present and not overlooked in the operative specimen [9].

IPAA is a complex procedure, requiring extensive expertise. High-volume operating teams achieve better results than surgeons performing IPAA sporadically [10]. Several complications may affect the outcome of surgery, pelvic sepsis being the most worrisome. Pelvic sepsis is reasonably considered the *bête noire *of IPAA [11], negatively impacting on function and HRQoL and potentially leading to failure, but recent advancements have led to a dramatic reduction of septic complications, currently happening in less than 25% of patients (7.7% failure) [12,13]. In our series, elderly patients were not at higher risk of sepsis. There still exists skepticism to recommend IPAA in the elderly. This may be justified by several reasons. First of all, evidences on IPAA in elderly patients are definitively lacking in the literature. Older patients are often found with comorbidities - as confirmed in our report - and this is believed to increase the risk of postoperative complications. There is a physiological decrease in sphincter function in older persons, which may lead to worse bowel control. These observations led some authors to recommend ileo-rectal anastomosis in elderly UC patients [14,15].

We are able to confute these points in the light of our results.

The lack of evidences is a fact. Farouk et al. [4] found patients undergoing IPAA in the elderly suffering from higher stool frequency, fecal incontinence, pad usage, need for constipating medication. Similar results were reported by Delaney et al [5], although differences seemed less apparent at 10-year follow-up. However, even if these are the most cited studies on the topic, due to the large number of observed patients, it should be remarked that they consider patients as "in the elderly" if over 45 or 50 years of age. We chose 70 years of age as a cut-off value, adjusting it on the basis of life expectancy, as it is more reliably including patients who are actually in an elderly condition (i.e. not able to cope with work activity), reflecting an higher rate of comorbidities and need for concomitant medications.

Comorbidities and concomitant medications may be an issue. In our series patients aged over 70 years were found with a significantly higher number of comorbidities, but this observation did not affect the outcome of surgery or HRQoL. Rather than considering comorbidities alone, one should assess general performance status, and offer IPAA only to suitable patients. The ideal candidate for IPAA should be autonomous, with no symptomatic sphincter disturbance, able to understand the risks and problems of the procedure, and willing to trade on surgery [2,4,5]. Older UC patients often need to take more than one drug [16] and are at risk of developing complications from drug interactions (i.e. azathioprine plus ACE-inhibitors increases the risk of leukopaenia and anemia [17]) and from prolonged consumption of medications (i.e. immunosuppressants and higher risk for malignancies [16,18]). IPAA has a protective effect on such complications, ensuring the avoidance of drugs to control UC-related symptoms. In our series, at 1-year follow-up elderly patients were more frequently taking antidiarrhoeals. Older patients may be used to "take drugs" to cope with disturbances or, more probably, with their fears. As long as they become conscious of their condition, and used to a new bowel functioning, a decrease or stabilization of antidiarrhoeals assumption is likely to be observed (table 3). Some peculiar aspects of elderly patients undergoing ileal diversion need to be discussed. We found a significant rate of dehydration in older patients with a stoma; one patient required readmission and intravenous fluid administration. Elderly patients may be under treatment with hypotensive agents preoperatively: reintroduction of the drugs is to be done carefully. Also, older individuals often have an impaired thirst mechanism. Dehydration must not be overlooked and should be promptly treated; electrolytes imbalance may potentially lead to dramatic consequences. Patients should be aware of the risk. Ileostomy takedown should be performed as soon as possible, taking into account the completeness of IPAA maturation period and patients' health status.

Continence tends to decrease in the elderly. However, it is important to investigate for symptomatic disturbances and not to overestimate them [19]. In details, patients with active or severe disease, with frequent emission of semi-liquid, hematic stools, may suffer from seepage and soiling. The frequent passage of irritating substances may cause this effect, and be misleading, and clinicians may overestimate the rate of incontinence: when the disease is controlled, continence is promptly restored. This contingent stool loss must be differentiated from actual impairment of continence, and an accurate examination by an expert surgeon allows for it. Also, when treating patients with lower life expectancy, the functional outcome might be more important than the potential long-term risk of malignancy: the preservation of the small rectal mucosa cuff and of the anal transitional zone by the double-stapled technique yields better functional outcomes and is to be preferred. This is the ideal situation for making an anastomosis at the upper canal level - where there may be better function.

## Conclusions

When dealing with older patients, it should always been considered that a well-functioning ileostomy may be preferable over an IPAA with poor functional outcome and incontinence. *IPAA should be offered selectively to elderly UC patients who are strongly motivated and with no clinical disturbances of continence. When these conditions are respected, no differences are observed in terms of complications and HRQoL. Once indications to surgery are established, IPAA is to be encouraged in suitable patients, as delaying the operation may result in sub-optimal outcomes, irrespective of age*.

In our series we found IPAA *safe *and with *excellent functional results *in *selected *elderly patients. Results are *stable *with time. Function and quality of life are comparable to those of younger patients.

## List of abbreviations used

IPAA: ileo-pouch-anal anastomosis; UC: ulcerative colitis; IBDQ: Inflammatory Bowel Disease Questionnaire; HRQoL: health-related quality of life; SD: standard deviation; ASA: American Society of Anesthesiologists; ACE-inhibitor: angiotensin-converting-enzyme inhibitor.

## Competing interests

The authors declare that they have no competing interests.

## Authors' contributions

G.P.: conception and design, interpetration of data, given final approval of the version to be published

G.S.: acquisition of data, drafting the manuscript, given final approval of the version to be published

G.C.: acquisition of data, drafting the manuscript, given final approval of the version to be published

A.C.: acquisition of data, drafting the manuscript, given final approval of the version to be published

R.M.: acquisition of data, drafting the manuscript, given final approval of the version to be published

F.R.: acquisition of data, drafting the manuscript, given final approval of the version to be published

S.D.F: acquisition of data, drafting the manuscript, given final approval of the version to be published

S.C.: critical revision, interpretation of data, given final approval of the version to be published

F.S.: conception and design, critical revision, given final approval of the version to be published

## Authors' information

GP: Resident in General Surgery at Second University of Naples.

GS: PhD, Researcher, Aggregate Professor of General Surgery at Second University of Naples.

GC: Resident in General Surgery at Second University of Naples.

AC: Medical Student (Research Assistant) at Second University of Naples.

RM: Medical Student (Research Assistant) at Second University of Naples.

FR: Medical Student (Research Assistant) at Second University of Naples.

SDF: M.D. (Research Assistant) at Second University of Naples.

CS: Full Professor of General Surgery at Second University of Naples, Chief Division of General and Geriatric Surgery Second University of Naples.

FS: Associate Professor of General Surgery at Second University of Naples.
